# Cell death regulation in myocardial toxicity induced by antineoplastic drugs

**DOI:** 10.3389/fcell.2023.1075917

**Published:** 2023-02-07

**Authors:** Xue Yu, Yan Yang, Tianzuo Chen, Yuqin Wang, Tianwei Guo, Yujun Liu, Hong Li, Liming Yang

**Affiliations:** ^1^ Department of Pathophysiology, School of Basic Medical Sciences, Harbin Medical University, Harbin, China; ^2^ Department of Pathophysiology, Harbin Medical University-Daqing, Daqing, China

**Keywords:** cardiotoxicity, doxorubicin, autophagy, apoptosis, ferroptosis, pyroptosis, necroptosis

## Abstract

Homeostatic regulation of cardiomyocytes plays a critical role in maintaining normal physiological activity of cardiac tissue. Severe cardiotoxicity can lead to heart disease, including but not limited to arrhythmias, myocardial infarction and cardiac hypertrophy. In recent years, significant progress has been made in developing new therapies for cancer that have dramatically changed the treatment of several malignancies and continue to improve patient survival, but can also lead to serious cardiac adverse effects. Mitochondria are key organelles that maintain homeostasis in myocardial tissue and have been extensively involved in various cardiovascular disease episodes, including ischemic cardiomyopathy, heart failure and stroke. Several studies support that mitochondrial targeting is a major determinant of the cardiotoxic effects triggered by chemotherapeutic agents increasingly used in solid and hematologic tumors. This antineoplastic therapy-induced mitochondrial toxicity is due to different mechanisms, usually altering the mitochondrial respiratory chain, energy production and mitochondrial kinetics, or inducing mitochondrial oxidative/nitrosative stress, ultimately leading to cell death. This review focuses on recent advances in forms of cardiac cell death and related mechanisms of antineoplastic drug-induced cardiotoxicity, including autophagy, ferroptosis, apoptosis, pyroptosis, and necroptosis, explores and evaluates key proteins involved in cardiac cell death signaling, and presents recent advances in cardioprotective strategies for this disease. It aims to provide theoretical basis and targets for the prevention and treatment of pharmacological cardiotoxicity in clinical settings.

## 1 Introduction

Advances in cancer treatment have significantly improved survival rates for cancer patients (Harake et al., 2012; Miller et al., 2019). There is, however, a high incidence of treatment-related morbidity associated with high patient survival rates. Modern cancer treatment has shown that drug combinations can lead to synergistic side effects, especially cardiovascular disease (Minotti et al., 2004). Cardiotoxicity is usually defined as toxicity that negatively affects the heart and can lead to cardiomyopathies such as arrhythmias, myocardial infarction, and cardiomyopathy (Ewer and Ewer, 2015a). Cardiotoxicity caused by antineoplastic drugs is divided into two types: irreversible cardiotoxicity and reversible cardiotoxicity. Irreversible myocardial damage caused by anthracyclines is classified as type I, a class II adverse effect is caused by the human epidermal growth factor receptor 2 (HER2) inhibitors trastuzumab and bevacizumab, sunitinib and sorafenib (Ewer and Ewer, 2015b). There is no evidence that type II drugs cause myocardial necroptosis, but they can cause cardiomyocyte dysfunction. Cancer survivors treated with anthracyclines are significantly more likely to suffer from cardiovascular disease. There is currently only one drug approved by the US Food and Drug Administration (FDA) for the prevention of doxorubicin (DOX)-induced cardiotoxicity: dexrazoxane (Kolaric et al., 1995). However, it may be associated with a lower tumour response to DOX and a higher risk of secondary malignancies (Swain et al., 1997), which severely limits its use in clinical practice. Despite extensive research on cardiotoxicity caused by antineoplastic drugs, the molecular pathogenesis remains unknown.

Mitochondria are important organelles in maintaining myocardial homeostasis, are involved in several cellular functions *in vivo* and regulate cell survival and death. The role of mitochondria in antineoplastic drug-induced myocardial toxicity has also received increasing attention in recent years (Zamorano et al., 2016). The clinical presentation is usually dose-dependent cardiomyopathy, which progresses to chronic heart failure with high morbidity and mortality (Natarajan et al., 2020). The evaluation of multifactorial processes during antineoplastic therapy based on the interaction of genetic and environmental factors, as well as a better understanding of the potential mechanisms of antineoplastic cardiotoxicity and adverse cardiovascular events, remains a major challenge in the field of cardiology. Here we review the role of cell death pathways in autophagy, apoptosis, ferroptosis, pyroptosis and necroptosis in oncological heart disease, and also present an update on current clinical research in this disease, focusing on strategies targeting mitochondria, We discuss the implications of these findings for understanding the molecular mechanisms underlying tumorigenic heart disease and identifying novel therapeutic targets.

## 2 Antineoplastic drug-induced cardiotoxicity

### 2.1 Doxorubicin

Anthracyclines are a class of drugs derived from *Streptomyces* anthracis and used in cancer chemotherapy (Martins-Teixeira and Carvalho, 2020). DOX, erythromycin, and epirubicin are the main anthracyclines approved for clinical use by the FDA. *Streptomyces* cecum, for example, produced DOX and erythromycin in the early 1960s. Although all anthracyclines have glycosidic units, the presence of methyl or alcohol groups in their structure gives a different spectrum of anticancer activity. They inhibit DNA replication and transcription, which in turn inhibit cell proliferation (Marinello et al., 2018; Hulst et al., 2022). Anthracyclines cause variable cardiotoxicity, with DOX causing the greatest cardiotoxicity. Therefore, we focused on DOX, which has the most significant and best known cardiotoxicity. The chemotherapeutic agent DOX can cause cardiotoxicity that leads to a chronic, progressive, and potentially fatal cardiomyopathy called doxorubicin-induced cardiomyopathy (DIC), a fatal cardiomyopathy with a poor prognosis that causes cardiotoxicity and limits the efficacy of doxorubicin in the treatment of malignancies. Clinically, DIC is characterised by decreased left ventricular ejection fraction, increased ventricular wall thickness, arrhythmias and potentially fatal heart failure (Henriksen, 2018).

### 2.2 Trastuzumab

There is only one FDA-approved therapeutic antibody for HER2-positive breast cancer, trastuzumab, which is an antibody against HER2 and an inhibitor of DNA topoisomerase I (Keam, 2020). In terms of further treatment, the ADC trastuzumab (T-DM1) and the HER2 kinase inhibitor lapatinib are currently approved (Geyer et al., 2006; Verma et al., 2012). First-line treatment for metastatic gastric cancer with HER2-positive cells is trastuzumab in combination with chemotherapy (Bang et al., 2010). Non-small cell lung cancer (Mazières et al., 2013), colorectal cancer (Siena et al., 2018) and biliary tract cancer (Nam et al., 2016) have been associated with HER2 overexpression and mutations. For solid tumours expressing HER2, however, no HER2-targeted therapies are approved. The humanised monoclonal antibody trastuzumab inhibits HER2. Combining trastuzumab with conventional chemotherapy improves survival in patients with metastatic or early-stage HER2-positive (HER2+) breast cancer (Barish et al., 2019). After 11 years of follow-up of a randomly selected cohort of 5,099 patients randomly assigned to the HERA trial, the group treated with trastuzumab for 1 year had a significantly lower risk of disease-free survival and death than the observation group. In all groups, cardiotoxicity was low and mostly occurred during treatment (Cameron et al., 2017). Cardiomyopathy caused by trastuzumab adversely affects both cardiac and tumour outcomes. Cardiological oncology collaborative studies have focused on risk stratification, early diagnosis, and prevention strategies for trastuzumab-induced cardiomyopathy.

### 2.3 The cardiotoxicity of doxorubicin and trastuzumab

Dox has been reported to cause dose-dependent myocardial damage that can be fatal in certain studies. Within a year of treatment, DOX-induced cardiomyopathy typically results in a decrease in left ventricular ejection fraction (LVEF), and heart failure may occur as a result (Mordente et al., 2012; Seferović et al., 2019). About half of the patients die within 2 years in this case (Govender et al., 2014). In their study, Mitry and colleagues found a correlation between heart failure and cumulative DOX exposure: the risk of heart failure is 4% at cumulative doses below 500 mg/m^2^; this risk increases to 36% at cumulative doses above 600 mg/m^2^ (Mitry and Edwards, 2016).

According to another study, the incidence of heart failure was only 0.27% among patients receiving less than 550 mg/m^2^ and 30% among patients receiving more than 550 mg/m^2^. According to the above results, the maximum cumulative lifetime dose should not exceed 400–450 mg/m^2^, and when this level is exceeded, the risk of cardiotoxicity increases significantly (Vejpongsa and Yeh, 2014). However, anthracycline doses below this level may still cause cardiotoxicity (Khanna et al., 2019). One study showed cardiotoxicity when anthracyclines are administered at doses up to 250 mg/m^2^ (Vandecruys et al., 2012). A subgroup of childhood cancer survivors exposed to very low anthracycline doses (100 mg/m^2^), subclinical abnormalities in left ventricular structure were observed by Leger (Leger et al., 2015). Although many studies have been conducted on anthracycline dosing. Further studies are needed to elucidate the mechanisms of cell death that cause myocardial toxicity.

Many cancer survivors suffer from long-lasting side effects of treatment, and cardiovascular complications are a major problem in breast cancer, the most common cancer in women (Anjos et al., 2021). Combining trastuzumab with conventional chemotherapy increases survival of patients with metastatic or early-stage breast cancer that is HER2-positive (HER2+). In preliminary clinical trials in people with metastatic breast cancer, heart failure was associated with heart failure in 4% of people who received trastuzumab alone, but 27% of people treated with trastuzumab in combination with anthracycline had symptoms of heart failure (Keefe, 2002).

The exact mechanism of the effect of trastuzumab on HER2 is not known and may involve multiple cellular pathways, just as the effect of trastuzumab on cardiomyocytes is not known. A major role is played by HER2 and ErbB family tyrosine receptors in the development and proliferation of myocardial cells, and trastuzumab-induced inhibition of intracellular signalling may affect cell metabolism, leading to myocardial cell dysfunction, impaired cell proliferation and survival (Milano et al., 2014). Trastuzumab-induced cardiomyopathy was recently demonstrated to be primarily caused by mitochondrial dysfunction and altered cellular energy metabolism in a human stem cell cardiomyocyte model (Kitani et al., 2019).

### 2.4 The role of antineoplastic drugs such as cyclophosphamide and cisplatin

Anthracyclines are the best known of the chemotherapeutic agents that cause cardiotoxicity. In addition, alkylating drugs, including cisplatin, cyclophosphamide, ifosfamide, carmustine, chlormethine, busulfan, and mitomycin, are also linked with cardiac toxicity (Pai and Nahata, 2000). Cyclophosphamide is an alkylating, anticancer agent which was first characterized in experiments on rat tumors. It is an oxazaphosphorine-substituted nitrogen mustard, with strong cytotoxic and immunosuppressive activity (Kim and Chan, 2017). Cyclophosphamide-induced cardiac injury is dose-dependent, with the total dose of a single course of therapy being the best indicator of toxicity, and patients receiving >150 mg/kg or 1.55 g/m^2^/d are at higher risk of cardiotoxicity (Kusumoto et al., 2013). The dose-limiting factor during cyclophosphamide therapy is cardiotoxicity, which is irreversible (Kanda et al., 2001). Fatal cardiomyopathy has been reported in 2–17% of patients taking cyclophosphamide. This depends on the treatment regimen and specific patient population characteristics (Ishida et al., 2016). Cardiotoxicity has been shown to be a dose-limiting factor during cyclophosphamide therapy, and although the mechanisms of cyclophosphamide-induced cardiotoxicity are not fully understood, they are thought to include oxidative and nitrative stress (Ayza et al., 2020).

Cisplatin is an extremely effective chemotherapeutic agent, a platinum-based drug that is highly active against ovarian, cervical, testicular, bladder, lung, and solid tumors and is resistant to other treatments (Yousef et al., 2009). Cisplatin shows cytotoxic effects by cross-linking DNA with purine bases, leading to DNA damage and apoptosis in cancer cells (Dasari and Tchounwou, 2014). Several studies have shown that cisplatin treatment may cause cardiotoxicity (El-Awady el et al., 2011; Patanè, 2014). Heart failure, arrhythmias, myocardial infarction, pericarditis, myocarditis, and congestive cardiomyopathy have been defined as symptoms of cardiotoxicity caused by cisplatin chemotherapy (Patanè, 2014). The cardiotoxicity of cisplatin has limited its clinical use. The mechanism of cisplatin cardiotoxicity is completely unknown, and few studies have improved the cardiotoxicity of cisplatin despite clinical data demonstrating the cardiotoxic effects of cisplatin. Oxidative stress plays a key role in cisplatin-induced toxicity (Başak Türkmen et al., 2022). When used in combination with doxorubicin, cisplatin and cyclophosphamide showed antigenotoxic activity and reduced phagocytosis and the potential for cell death (Juliano Oliveira et al., 2019).

## 3 The cardiomyocyte death pathways of doxorubicin and trastuzumab

### 3.1 Autophagy

After being separated according to their damaged mitochondrial membrane potential, damaged mitochondria are engulfed by autophagosomes and transported to lysosomes for degradation (Thomas and Gustafsson, 2013; Park et al., 2021). ULK-1, an upstream signaling protein of autophagy, regulates the unc-51-like autophagy-activated kinase complex (ULK-1). Its regulation is tightly controlled by mammalian targets of the serine/threonine kinases AMP-activated protein kinase (AMPK) and rapamycin (mTOR), with AMPK activating and mTOR inhibiting ULK-1 (Koleini and Kardami, 2017). DOX, on the other hand, inhibits mTOR signaling while activating the phosphorylation of Beclin-1 *via* ULK-1, and phosphorylated Beclin1 initiates autophagy. This process is exacerbated further by increased AMPK phosphorylation, which activates the ULK-1 complex and Beclin1, and then activates vacuum protein sorting 34 (Vps34) and Vps15, a complex that mediates autophagosome formation and phagosome extension by recruiting more Atg proteins (Levine and Kroemer, 2019; Klionsky et al., 2021). The autophagosomal protein LC3-I lipidizes to form LC3-II and protein p62, which classifies proteins and others within the autophagosome (Zhang et al., 2021a). After fusion with the lysosome, the protease breaks down the autophagosome and explains or recycles its damaged components (Qiang et al., 2021; Russo et al., 2021).

DIC is a two-edged sword, as autophagy can either prevent cardiotoxicity or exacerbate the disease state if autophagy levels exceed a certain threshold (Wang et al., 2020). Furthermore, doxorubicin may induce or inhibit autophagy in cardiac tissue (Table 1). It has been shown that doxorubicin inhibits autophagic flux in cardiac myocytes by reducing lysosomal acidification and function, and that a reduction in autophagy prevents the cardiotoxic effect of doxorubicin. However, its subsequent inhibition helps to resolve this apparent contradiction (Bartlett et al., 2016; Li et al., 2016; Cao et al., 2017). Accumulation of uninterrupted autophagosomes and autolysosomes in cells exacerbates cardiomyocyte damage and even leads to cardiomyocyte death. In particular, low doses of doxorubicin increased the expression of LC3-II, p62 and Beclin1 proteins, suggesting that autophagy is induced (Li et al., 2016). However, in terms of autophagy downstream activity, doxorubicin inhibited autophagic flux and lysosomal acidification in cardiomyocytes. The accumulation of uninterrupted autolysosomes results in ROS production and DIC due to the inhibition of autophagy (Li et al., 2016).

**TABLE 1:** Evidence of doxorubicin-induced autophagy in the heart.

Model	Targets	Autophagy	Treatment/Effect on DIC	References
NRCM; C57BL/6J mice	AMPK ↑	Increase	Ghrelin/protection	Wang et al. (2014)
	LC3-II ↑		3-MA/protection	
	Atg 5, 6, 8, 12↑		Compound C/protection	
H9C2 cells, C57BL/6J mice	Beclin 1 ↑	Increase	Ophiopogonin-D/protection	Zhang et al. (2015)
	LC3II/I ↑		3-MA/protection	
NRCMs	GATA4 ↓	Increase	3-MA/protection	Kobayashi et al. (2010)
	Bcl-2 ↓		Rapamycin/susceptibility	
	LC3-II ↑ autophagy flux ↑			
C57BL/6 J mice; NRCM; H9C2	LC3-II ↑	Decrease	Beclin1^+/−^/protection	Li et al. (2016)
	Lysosomal cathepsins ↓		Beclin1 overexpression/susceptibility	
			BafA1/protection	
SD Rat; ARCM; AMCM; NRCM; H9C2	TFEB ↓	Decrease	TFEB overexpression/protection	Bartlett et al. (2016)
	Cathepsin B ↓		Torin 1/protection	
SD Rat; NRCM; H9C2	AMPK ↓ mTOR ↑	Decrease	Astragalus polysaccharides/protection	Cao et al. (2017)
	LC3-II ↓			
	Autophagic flux ↓			

NRCM, neonatal rat cardiomyocytes; ARCM, adult rat cardiomyocytes; 3-MA, 3-methyl adenine; BafA1, bafilomycin one; AMCM, adult mice cardiomyocytes; TFEB, transcription factor EB; LC3-II, microtubule-associated protein 1A/1B-light chain three; Atg, autophagy-related protein; SD, sprague dawley; ↓ decrease, ↑ increase.

When doxorubicin is administered to Beclin1 haploinsufficient mice with impaired autophagy initiation, the number of unprocessed autolysosomes is reduced compared to wild-type mice, leading to reduced ROS production and impaired DIC. In contrast, increased autophagy through overexpression of Beclin1 enhances DIC (Li et al., 2016). Similarly, doxorubicin inhibits the expression of transcription factor EB (TFEB), which in turn inhibits the hydrolysis of lysosomal proteins, leading to decreased autolysosome accumulation and viability (Bartlett et al., 2016). TFEB is a positive regulator of autophagy, involved in autophagosome processing and lysosome function. As a result of Torin-mediated gene repair and pharmacological activation of TFEB, doxorubicin-induced inhibition of histone B (lysosomal cysteine protease) was prevented and ROS production was increased (Bartlett et al., 2016). Recent studies have shown that doxorubicin inhibits lysosomal protein hydrolysis, Inducing autophagosomes and autolysosomes accumulation and ROS production. A potential treatment strategy is to inhibit autophagy or stimulate lysosomal function, which reduces autolysosome accumulation and ROS production (Table 1).

In addition, doxorubicin has been shown to enhance autophagy in cardiac myocytes in several studies. Xue Wang found that DOX exposure significantly increased AMPK, LC3-II expression in H9C2 cells, and enhanced autophagy paralleled severe apoptosis and size reduction in cardiac myocytes. Growth hormone-releasing peptide was found to reduce oxidative stress and leukophagy in mouse heart and H9C2 cells. DOX damage leads to reduced apoptosis, increased cell size and improved cardiac performance in cardiac myocytes (Wang et al., 2014). Another study demonstrated that DOX induced excessive autophagy through the generation of reactive oxygen species (ROS) in H9C2 cells and mouse hearts, as evidenced by a significant increase in the number of autophagic vesicles, LC3-II/LC3-I ratio, and upregulation of GFP-LC3 expression. Pretreatment with Ophiopogonin D partially attenuated the above phenomenon, similar to the effect of treatment with 3-methyladenine (Zhang et al., 2015). Satoru Kobayashi found that DOX significantly increased autophagic flux in cardiomyocytes, as indicated by differences in protein levels of LC3-II (microtubule-associated protein light chain 3 form 2) or the lysosomal inhibitor bafilomycin A1 for autophagy Differences in the number of vesicles are shown. DOX-induced cardiomyocyte death, as determined by multiple assays, was exacerbated by drugs or genetic approaches that activate autophagy but attenuated by manipulations that inhibit autophagy, suggesting that activation of autophagy mediates DOX cardiotoxicity and that preservation of the Transcription factor GATA4 attenuates DOX by regulating the expression of Bcl2 and autophagy-related genes to inhibit autophagy cardiotoxicity (Kobayashi et al., 2010) (Table 1).

### 3.2 Apoptosis

Apoptosis is the most common form of programmed cell death, characterized by cell shrinkage, increased cytoplasmic density, and loss of mitochondrial membrane potential. As a result of the changes in permeability, intact apoptotic bodies are generated, which neighboring cells efficiently take up and degrade (Bertheloot et al., 2021; Wang et al., 2021). Apoptosis is classified as intrinsic or extrinsic. The intrinsic pathway is activated by toxic substances or DNA damage that cause dysregulation or imbalance of intracellular homeostasis. This condition is characterized by increased permeability of the outer mitochondrial membrane, resulting in the release of cytochrome C. The release of mitochondria outer membrane permeability (MOMP) and cytochrome C causes the formation of apoptotic bodies and the activation of caspase-3. Intrinsic apoptosis is primarily regulated by its effect on mitochondria (Hutt, 2015). In contrast to intrinsic apoptosis, it is triggered by activation of cell surface death receptors. When pro-apoptotic death receptors are activated by their ligands, they form platforms on the cell surface. In turn, they recruit adapter proteins (TRADD and FADD) and activate the apoptosis promoters caspase-8 and -10 to induce apoptosis (Matsuda et al., 2014; Zheng and Kanneganti, 2020).

**FIGURE 1:**
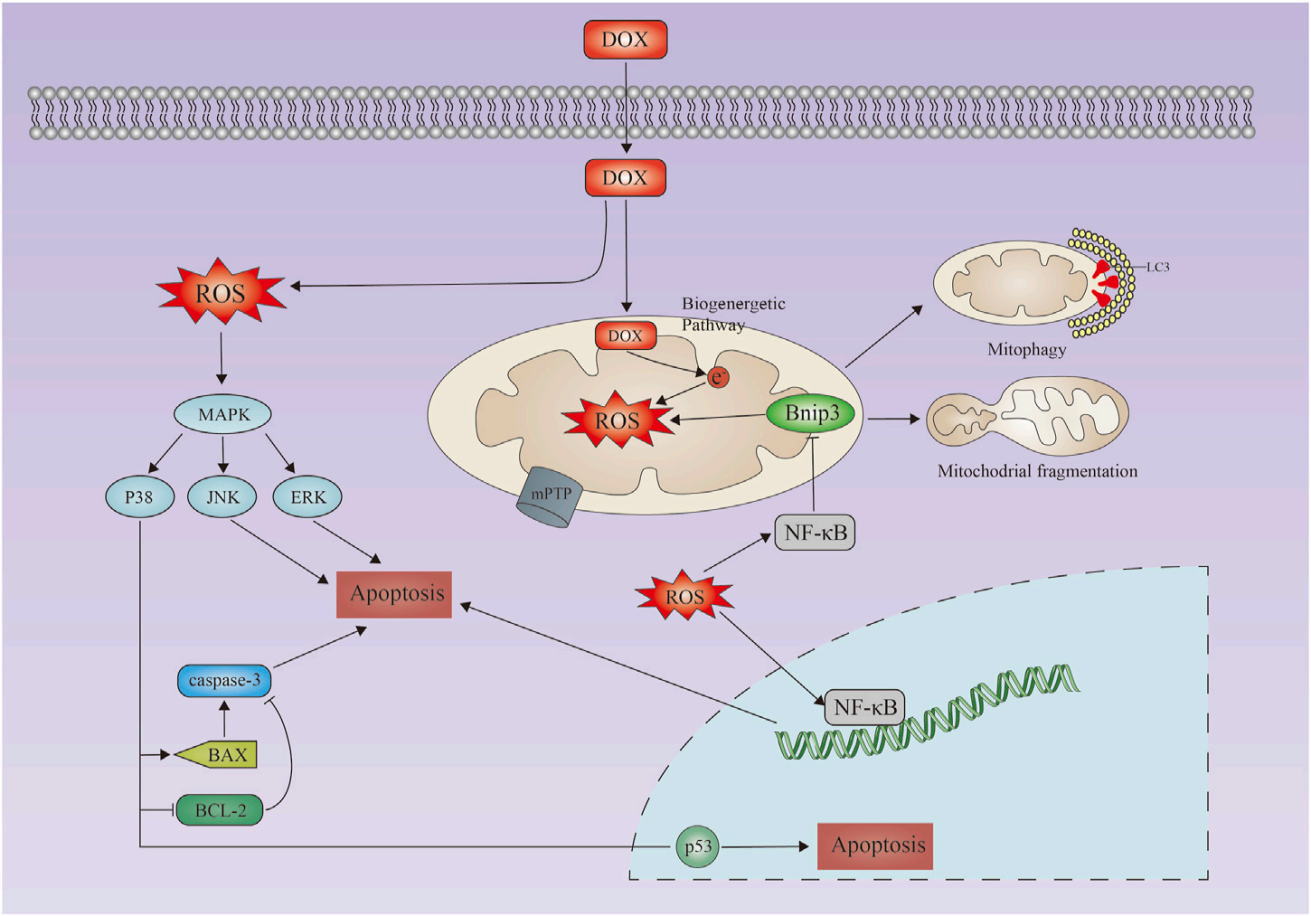
Doxorubicin-induced apoptosis in cardiacmyocytes. Schematic representation of doxorubicin-induced apoptosis in cardiac myocytes. Doxorubicin induces increased reactive oxygen species production, activation of the MAPK pathway, upregulation of Bax/Bak, and activation of caspases 3, leading to apoptosis. Mitochondrial calcium overload and activation of the mitochondrial permeability transition pore (mPTP) lead to loss of mitochondrial membrane potential, mitochondrial swelling, and outer membrane rupture. DOX, doxorubicin; MAPK, mitogen-activated protein kinase; JNK, Jun amino-terminal kinase; ERK, extracellular signal-regulated kinase.

Apoptosis is the best studied and characterized pathway of programmed cell death (Figure 1). The treatment with doxorubicin induces excessive oxidative stress and mitochondrial damage, which activates apoptosis in cells (Wenningmann et al., 2019). Induced mitochondrial permeability due to doxorubicin can activate the intrinsic apoptosis pathway, which results in the proliferation of the proapoptotic factor cytochrome C (An et al., 2009). Doxorubicin induces intrinsic apoptosis through several mechanisms, including activation of p53 leading to Bax activation, downregulation of GATA4 (L'Ecuyer et al., 2006), which reduces the activation of the anti-apoptotic Bcl-XL expressing JNK and MAPK, and inactivation of the PI-3K/Akt-prospurvival pathway. In cardiomyocytes, doxorubicin also induces exogenous apoptosis pathways (Nakamura et al., 2000). A death ligand such as FasL or TNF binds to its receptor and recruits Fas-associated proteins to the cytoplasm *via* the FADD (death architecture domain) and the TRADD (death architecture domain) and TRADD (TNFR-related death architecture domain) (Kalivendi et al., 2005; Shi et al., 2011). Induced activation of caspase-8 by FADD and TRADD activates caspase-3, which leads to apoptosis (Lavrik and Krammer, 2012). Doxorubicin also promotes an extrinsic apoptosis pathway. This causes an upregulation of Fas/FasL and p53 and a downregulation of the FLICE/caspase-8 inhibitor protein FLIP after activating nuclear factor-activated T cells-4 (Minotti et al., 2001; Nitobe et al., 2003; Shati, 2020) (Figure 1).

Transtuzumab alters the expression of TopII genes and proteins in cardiomyocytes, leading to apoptosis, is one reason why anthracyclines and trastuzumab together increase the risk of cardiotoxicity. It involves cellular processing with little to no inflammation in surrounding tissues. In the physiological state, apoptosis regulates the development of cardiomyocytes and the stability of the cardiac internal environment (Loreto et al., 2014). In spite of this, apoptotic dysregulation plays an important role in cardiac remodeling and left ventricular dysfunction, which are defining characteristics of DIC. Intrinsic and extrinsic signaling pathways are the two most common signaling pathways that drive apoptosis.

The primary mechanism of DOX-induced cardiotoxicity is mitochondrial oxidative stress (Rabelo et al., 2001; Wattanapitayakul et al., 2005), and N-acetylcysteine and resveratrol have been reported to be effective strategies to reduce DOX-induced cardiotoxicity (Burridge et al., 2016; Abe et al., 2018). As a result of DOX administration, the major glycolytic enzymes, triphosphate isomerase, enolase, and ubiquinone oxidoreductases (which act as electron transport proteins in the mitochondrial respiratory chain), are oxidized in the heart and their activity decreases. The bioenergetic pathway may be a target of DOX-induced oxidative stress (Chen et al., 2006). The mitochondria are a source and a target of oxidative stress, according to many studies (Ascensão et al., 2011). DOX-induced cardiac dysfunction, mitochondrial damage, protein nitration and apoptosis were significantly worse in hearts from mice lacking glutathione peroxidase (GPX) than in hearts from wild-type mice (Gao et al., 2008). Although pretreatment of juveniles with oxidative stress stimulants may enhance antioxidant stress mechanisms in cardiomyocytes. Similar cellular responses are expected in cancer cells, which makes clinical application difficult (Figure 1).

DOX exerts its toxic effects through oxidative stress, but an emerging mechanism is endoplasmic reticulum (ER) stress, whose activation involves a pro-apoptotic pathway of the protein kinase RNA (PKR)-like ER kinase (PERK)/activated transcription factor-4 (ATF4)/C/EBP homologous protein (CHOP) axis. These stresses lead to myocardial dysfunction associated with cell death. Although a growing body of evidence supports their association with DOX-induced cardiotoxicity, the mechanisms have not been well elucidated (Kim et al., 2022). It has been suggested that mitochondrial and ER stress play an integral role in DOX-induced cardiotoxicity through interactions in which inhibition of DNA damage-induced transcript-3 (DDIT3) or calnexin is also critical for achieving Dox resistance in cardiomyocytes (Bagchi et al., 2021). The increase in DDIT3 found in DOX-treated cardiomyocytes for 24 h suggests that the increase in MitoBax may promote ER stress-related changes in DDIT3, compared to breast cancer MCF7 cells that show increased DDIT3 in response to ER stress in response to DOX as early as 3 h (Bagchi et al., 2021). If ER stress persists or is exacerbated, cancer cells are unable to re-establish ER homeostasis through ER-specific unfolded protein response (UPR), and ER stress shifts from a pro-survival to a pro-apoptotic state (Ferri and Kroemer, 2001). Therefore, promoting ER stress to initiate apoptotic pathways may be a therapeutic strategy for anticancer activity (Tabas and Ron, 2011). Prolonged activation of inositol-requiring protein-1 (IRE1) and C/EBP homologous protein (CHOP) can trigger apoptosis under certain physiological and pathophysiological conditions (Szegezdi et al., 2006). Experiments have shown that mammalian IRE1α binds Bak and Bax, proteins involved in the mitochondrial pathway of apoptosis. This interaction appears to be important for IRE1α activation (Hetz et al., 2006). Studies using shredded null mice have identified a role for CHOP in ER stress-induced apoptosis in many disease models, including renal insufficiency (Zinszner et al., 1998), advanced atherosclerosis (Tsukano et al., 2010), and cardiac pressure overload (Fu et al., 2010). One of the more widely cited mechanisms of CHOP-induced apoptosis is inhibition of the pro-survival protein Bcl-2, originally based on a study that showed the relevance of CHOP expression, oxidative stress, apoptosis, and Bcl-2 in CHOP-transfected rat fibroblast cell lines (McCullough et al., 2001). Most importantly, genetic recovery of Bcl-2 rescued CHOP-transfected cells from oxidative stress and apoptosis. The mechanism may involve the ability of CHOP to interact with one or more transcriptional repressors to reduce Bcl2 transcription (McCullough et al., 2001).

It has also been shown that DOX-mediated cardiomyocyte apoptosis is associated with the Hippo-YAP signaling pathway. DOX-induced cardiotoxicity is mediated by vascular injury, resulting in reduced cardiac blood flow and leading to cardiomyocyte apoptosis through activation of Hippo-YAP signaling. Furthermore, exercise (Ex) inhibits these effects by promoting the migration of BM stem cells to the heart to repair cardiac vessels damaged by DOX and by inhibiting DOX-induced Hippo-YAP signaling-mediated apoptosis. These data support the concept of using exercise as an intervention to reduce DOX-induced cardiotoxicity (Tao et al., 2021). Furthermore, DOX treatment successfully induced Akt/glycogen synthase kinase-3β (Gsk3β) inactivation *via* Hippo signaling pathway activation and promoted YAP degradation, thereby inhibiting colorectal tumorigenesis (Hu et al., 2023). It has also been shown that RASSF6 is downregulated in human bladder cancer and regulates DOX sensitivity and mitochondrial membrane potential through the hippocampal signaling pathway (Tan et al., 2019). RASSF6 belongs to the RASSF family with a Ras-associated structural domain, which has been reported to be involved in the Hippo signaling pathway. RASSF6 overexpression was found to affect the Hippo signaling pathway by downregulating YAP. depletion of YAP downregulated Bcl-xL expression and abolished the effect of RASSF6 on Bcl-xL. YAP depletion also upregulated the level of apoptosis and downregulated mitochondrial membrane potential. Yap siRNA abrogated the effect of RASSF6 on DOX-induced apoptosis and loss of mitochondrial membrane potential (Tan et al., 2019). Thus the Hippo signaling pathway is also an important target for attenuating DOX-mediated apoptosis in cardiomyocytes.

Cisplatin is a chemotherapeutic agent used in several cancers, and cisplatin-treated cardiomyocytes show mitochondrial abnormalities such as mitochondrial membrane depolarization, increased inflammatory response and ER stress, which ultimately stimulate cystein-3 activity and induce apoptosis (Chowdhury et al., 2016). In addition, emerging evidence suggests a close link between oxidative stress and cisplatin-induced apoptosis in cardiomyocytes. El-Awady el et al. found that cisplatin ameliorated lipid peroxidation, decreased GSH content and inhibited SOD activity, implying cisplatin-induced oxidative stress (El-Awady el et al., 2011). In addition, mitochondrial DNA damage and nuclear DNA damage were also observed. Antioxidant natural products such as tutin (vitamin P1), gingerone and anthocyanins inhibited cisplatin-induced inflammatory infiltration, DNA damage and mitochondrial dysfunction, suggesting a key role of oxidative stress in cisplatin-induced cardiomyocyte apoptosis (Qian et al., 2018; Soliman et al., 2018; Topal et al., 2018). Cyclophosphamide is commonly used in the treatment of malignancies such as leukemia and lymphoma, and also as an immunosuppressant for the treatment of systemic lupus erythematosus and polymyositis. Due to the dose-dependent approach, cyclophosphamide induced cardiotoxicity is largely consistent with high-dose therapy (Nishikawa et al., 2015). Acrolein, the active metabolite of cyclophosphamide, has been shown to be a major cause of cardiomyocyte death (Kurauchi et al., 2017). Cardiomyocyte injury induced by cyclophosphamide treatment includes sarcoplasmic reticulum expansion, mitochondrial disruption, and nuclear membrane invagination (Lushnikova et al., 2008). Further studies attributed these injuries to oxidative stress, elucidating that acrolein causes oxidative and nitrosative stress by inhibiting intracellular GSH and SOD and increasing MDA (Omole et al., 2018). Corresponding lipid peroxidation initiates impairment of mitochondrial function, which further leads to collapse of APT production and activation of cystein-3, leading to apoptosis (Refaie et al., 2020). In addition, cyclophosphamide was verified to stimulate TLR4, *via* TLR4/NF-κB signaling to trigger an inflammatory response and ultimately apoptosis (El-Agamy et al., 2017).

### 3.3 Ferroptosis

**FIGURE 2:**
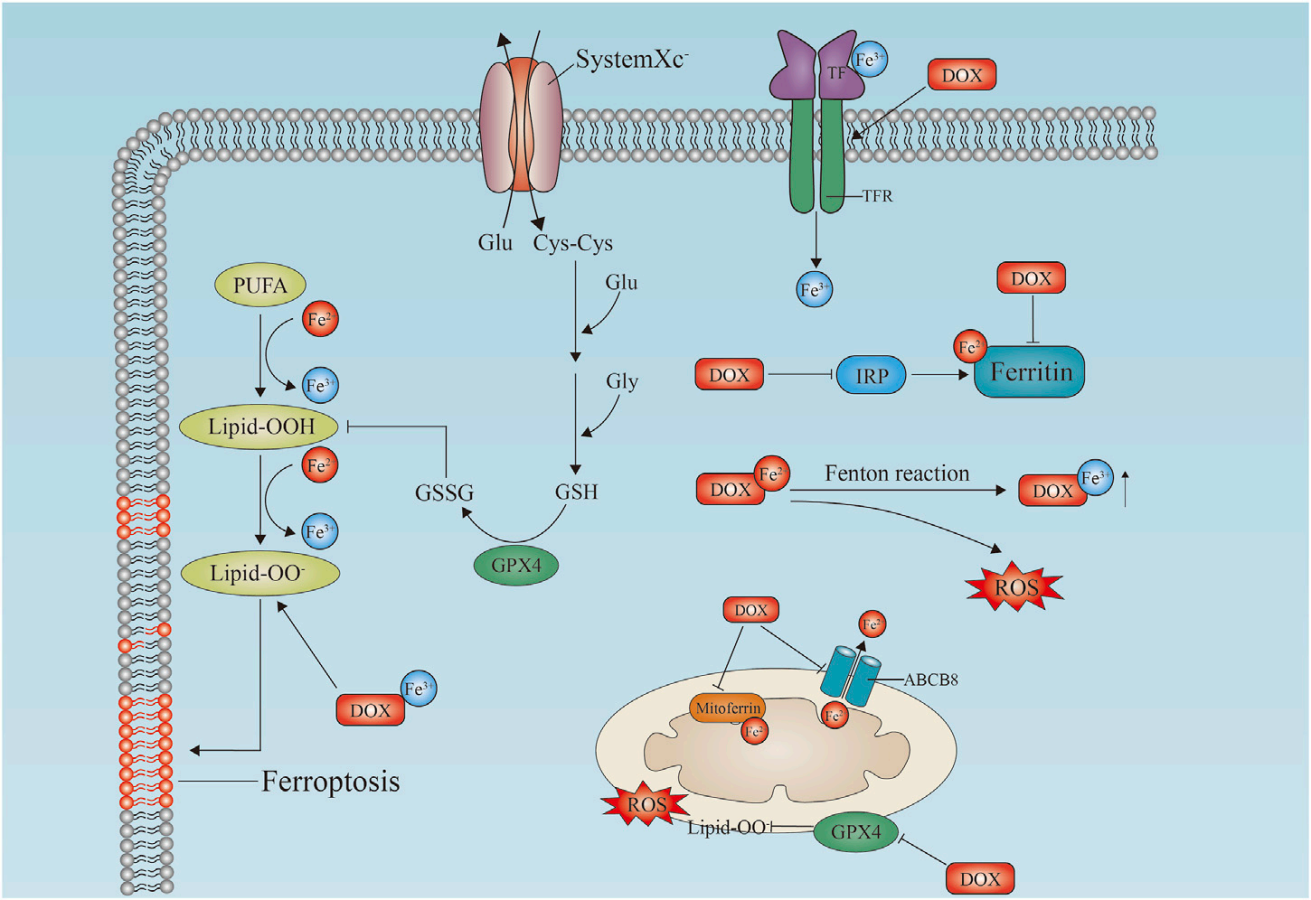
Doxorubicin-induced ferroptosis in cardiomyocytes. Schematic diagram of the doxorubicin-induced ferroptosis pathway in cardiomyocytes. Doxorubicin upregulates TfR and inactivates ferritin, which induces lipid peroxidation by inhibiting GPX4 in cell membranes and mitochondria, leading to ferroptosis. Free iron binds to doxorubicin to generate reactive oxygen species (ROS). In mitochondria, doxorubicin causes iron overload by blocking MitoFer and ABCB8. IRP, iron response regulatory protein; Tf, transferrin; TfR, transferrin receptor; Lipid-OO, lipid peroxidation; PUFA, polyunsaturated fatty acid; GSH, reduced glutathione; GSSG, glutathione disulfide; GPX4, glutathione Peroxidase; MitoFerrin, mitochondria ferritin; ABCB8, ATP-binding cassette transporter eight; ROS, reactive oxygen species; DOX, doxorubicin.

Iron-dependent accumulation of lipid peroxide is the hallmark of ferroptosis, a recently identified type of cell death, and ferroptosis is another mechanism of cardiac cell death through which doxorubicin induces cardiotoxicity (Fang et al., 2019; Su et al., 2020; Chen et al., 2021; Koppula et al., 2021) (Figure 2). ROS are produced by lipid peroxides, and iron plays a critical role in DIC. Doxorubicin treatment increases the toxic levels of unstable iron in cells. Doxorubicin interferes with ferritin iron regulatory protein (IRE) mRNA, resulting in decreased ferritin levels and increased levels of unstable iron (Canzoneri and Oyelere, 2008). In a similar manner, excess iron is released into the cell as a result of an increased amount of iron released by doxorubicin transferrin receptors (TfRs). Consequently, inhibition of TfR reduces iron uptake, intracellular oxidant formation, and cell death (Kotamraju et al., 2002; He et al., 2021). Kai Hou and colleagues studied the effects of TRIM21 in TRIM21 knockout mice in a doxorubicin treatment model and a left anterior descending branch (LAD)-induced cardiotoxicity model and found that TRIM21 knockout mice were protected from heart failure and death in both models. The hearts of wild-type mice treated with doxorubicin showed malformed mitochondria and increased lipid peroxidation and condensation of produced iron, which were attenuated in TRIM21 knockout mice. Mechanistically, in TRIM21-deficient heart tissue, Keap1 secretion by p62 is increased and protected from doxorubicin-induced ferritin. Reconstitution of TRIM21 mutants deficient in wild-type but not in p62 E3 ligase and death binding would prevent protection from doxorubicin-induced cell death (Hou et al., 2021).

By inhibiting glutathione peroxidase 4 (GPX4), doxorubicin causes excessive lipid peroxidation in mitochondria *via* the DOX-Fe^2+^ complex. The mitochondria-dependent ferroptosis that leads to DIC is caused by mitochondria-dependent ferroptosis (Tadokoro et al., 2020). As a result of the activation of nuclear factor red lineage two related factor 2 (NRF-2), doxorubicin induces ferroptosis in mice by increasing the expression of heme oxygenase 1 (hmox1) (Fang et al., 2019). Heme degradation is catalyzed by Hmox1, leading to oxidized lipid accumulation in mitochondrial membranes and free iron release. As well as inhibiting the antiferritin protein glutathione peroxidase 4 (GPX4) in the mitochondria and cytoplasm, doxorubicin-Fe induces excessive lipid peroxidation (Tadokoro et al., 2020; Wang et al., 2022a) (Figure 2).

In doxorubicin-induced ferroptosis, mitochondria play a critical role in ferroptosis. This evidence highlights the importance of ferroptosis in DIC (Liu et al., 2020). MitoTempo, a mitochondrial antioxidant that completely inhibits ferroptosis (Fang et al., 2019), and ferroinhibitor-1 may be promising cardioprotective agents to mitigate the cardiotoxic effects of doxorubicin (Xue et al., 2020; Zhang et al., 2021b; Jiang et al., 2021). Lei Sun and colleagues found that Fer-1 reversed the trastuzumab-induced decrease in cell viability, GSH/GSSG ratio, mitochondrial membrane potential and ATP content in a dose- and time-dependent manner. Fer-1 also reversed the effect of trastuzumab on GPX4, mitochondrial optical atrophy 1–1/2 and nematocystin expression levels. Trastuzumab-induced increases in mitochondrial ROS and iron levels were reversed by Fer-1 in H9C2 cells, and levels of acyl coenzyme A protein expression were increased as well (Sun et al., 2022). Shengting Wang and colleagues found that cir-BGN levels were significantly higher in trastuzumab-resistant breast cancer cells and tissues, which was associated with poor overall survival. cir-BGN degradation reduced the viability of breast cancer cells and significantly restored their sensitivity to trastuzumab. Erastin, a small molecule ferroptosis inducer, effectively restored the antitumor effect of trastuzumab, and the results suggest a novel circRNA that controls trastuzumab resistance by regulating ferroptosis. This provides a new treatment strategy and research platform for trastuzumab-resistant breast cancer patients (Wang et al., 2022b).

### 3.4 Pyroptosis

**FIGURE 3:**
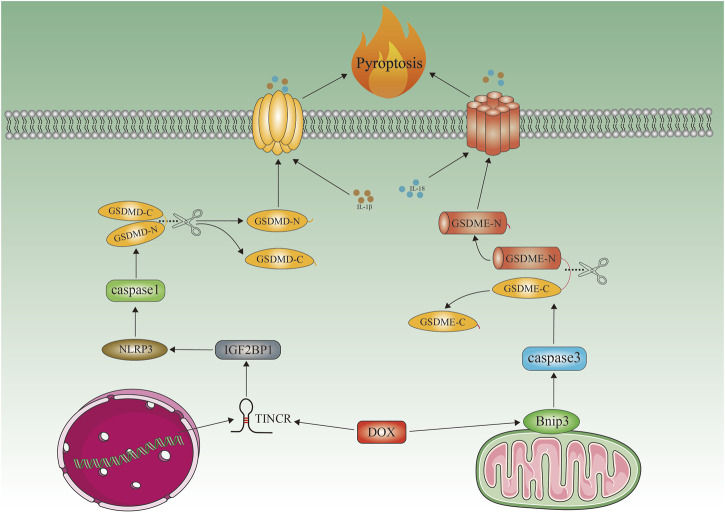
Doxorubicin-induced cardiomyocyte pyroptosis. Schematic representation of doxorubicin-induced cardiomyocyte pyroptosis. Doxorubicin recruits IGF2BP and increases NLRP3 expression by upregulating TINCR, which in turn activates caspase1, cleaves GMDSD-N, and releases IL-1β, IL-18. Doxorubicin activates Bnip3 in mitochondria, which activates caspase3 and causes GSDME-dependent pyroptosis. Bnip3, BCL2-interacting protein three; TINCR, terminal differentiation-induced NcRNA; NLRP3, NOD-, LRR-, and pyrin domain-containing protein three; GSDME, gasdermin E; GSDMD, gasdermin D; IGF2BP1, insulin-like growth factor 2 mRNA-binding protein 1.

A key player in cardiovascular diseases is now widely recognized as pyroptosis, discovered in 2001 (Jia et al., 2019; Yu et al., 2021). Inflammation of caspase-1, caspase-3, and caspase-11, as well as increased and activated NLR-containing family 3 (NLRP3) pyridine structural domains are characteristic of Pyroptosis, which leads to cleavage of hasdermin D (GSDMD) or GSDME and plasma membrane rupture, allowing release of interleukin-1 (IL-1) and IL-18 (Ruan et al., 2020; Zeng et al., 2020). Doxorubicin-induced pyroptosis is characterized by increased NLRP3 expression, IGF2BP recruitment, caspase-1 activation, GMDSD-N cleavage, and IL-1 and IL-18 release (Meng et al., 2019) (Figure 3). In contrast, MCC950 protects cells from doxorubicin-induced cell death (Meng et al., 2019). It has also been demonstrated that doxorubicin mediates pyroptosis in mitochondria *via* the activation of Bnip3 (Zheng et al., 2020). Protection against DIC has also been demonstrated with embryonic stem cell-derived exosomes by blocking the NLRP3/caspase-1 marker. The overexpression of heat shock protein 22 and the inhibition of NLRP3 by pharmacological agents (Tavakoli Dargani and Singla, 2019). By activating the NRF-2/SIRT3 signaling pathway, Gu and colleagues found that PCT inhibited NLRP3 and protected cardiomyocytes from doxorubicin-induced pyroptosis damage (Gu et al., 2021). NLRP3 was inhibited by sirtuin one activation and cardiomyocytes were protected from doxorubicin-induced pyroptosis (Sun et al., 2020). There may be a limit to new strategies against DIC if pyroptosis-associated molecules such as NLRP3, caspase-1, and Bnip3 are inhibited (Figure 3).

### 3.5 Necroptosis

In addition to necroptosis, doxorubicin also causes a form of necrosis known as necrosis that is controlled by signaling molecules called cytokines (Linkermann and Green, 2014). It is similar in mechanics to apoptosis and morphologically to necroptosis (Christofferson and Yuan, 2010). The necroptosis cascade is mediated primarily by the receptor-like cytoplasmic kinase1 (RIPK1), RIPK3 and mixed-spectrum kinase structural domain-like pseudokinase (MLKL), while necroptosis inhibitor-1 (Nec-1) inhibits necroptosis. The first inhibitor known to specifically block RIPK1 in necroptosis (Degterev et al., 2008). Pattern recognition receptors (PRRs), tumour necrosis factor receptor (TNFR) superfamily members, and other stimuli can activate the necroptotic cell death pathway. The TCRs on T cells and several chemotherapeutic agents play a key role in cancer (Lalaoui et al., 2015). The tumor necrosis factor (TNF)/TNFR pathway is considered a prototype among the various stimuli and has received the most attention (Fulda, 2013). Thus, events in the TNF signalling pathway may be focused on the onset of necroptosis. TNF- activates the TNFR1-associated death domain (TRADD) protein *via* TRFR1, which then phosphorylates RIPK1. To form necrosomes, it recruits and phosphorylates RIPK3 (Li et al., 2012; Choi et al., 2019). The necrosomes then phosphorylate a structural protein resembling the structural domain-like protein (MLK1) of mixed protein kinase. An immune response is triggered by the release of organelles and inflammatory factors, which results in the death of cells (Linkermann and Green, 2014).

Researchers have found that left ventricular samples from patients with end-stage heart failure have increased expression of necroptosis proteins, suggesting that this disease may contribute to heart failure (Szobi et al., 2017). Notably, necroinhibitor-1 is protective *in vitro* in DIC. It is suggested that both apoptosis and necroptosis are involved in the pathogenesis of DIC when dexrazoxane is used in conjunction with doxorubicin treatment (Yu et al., 2020). Evidence suggests that high doses or prolonged exposure to DOX treatment induces necroptosis of cardiomyocytes, instead of apoptosis and autophagy. The accumulation of ROS and peroxynitrite and the dose-dependent increase in DOX increase the death rate of cardiomyocytes during necroptosis (Fulbright et al., 2015). DOX is usually administered at a dose of 20 mg/kg *in vivo* and 1 μM *in vitro*. In mice, a dose of 25 mg/kg DOX can cause immediate necroptosis and heart failure when injected intraperitoneally once (Li et al., 2014), and 2 μM DOX can directly induce necroptosis of cardiomyocytes *in vitro* (Bernuzzi et al., 2009). When cardiomyocytes are exposed to DOX for a prolonged period of time, initial apoptosis evolves into necroptosis, with cells preferentially exhibiting early DNA damage and nuclear swelling (Rharass et al., 2016). Moreover, doxorubicin induces necroptosis through alternative and neo-die cellular pathways, resulting in more cell death than apoptosis (Zhang et al., 2016). Furthermore, Ting Zhang discovered that doxorubicin activates RIPK3 to regulate the opening of the mitochondrial permeability transition pore (mPTP), and promoting the binding and phosphorylation of RIPK3 with calmodulin kinase II (CaMKII), leading to apoptosis and necroptosis. Necroptosis also appears to be possible in the absence of RIPK1 and MLKL (Zhang et al., 2016). A better understanding of the specific participants in the doxorubicin-induced necroptosis process will allow new drugs to be identified that may stop this process. A CAMKII inhibitor, KN-93, and necroinhibitor-1 have been shown to protect against DIC in experimental models (Zhang et al., 2016). As previously stated, oxidative stress can disrupt lysosomal function and normal autophagy. As a result, delayed autophagy causes more severe apoptosis secondary to necroptosis in cardiomyocytes (Dimitrakis et al., 2012; Li et al., 2014). These findings lend support to the theory that necroptosis occurs after prolonged exposure to DOX treatment.

## 4 Outlook

Cardiotoxicity caused by common anticancer drugs remains a major clinical problem that can affect the quality of life and overall survival of cancer patients. Although the mechanisms underlying cardiotoxicity of antineoplastic drugs are multifactorial and appear to involve several different pathways, there is increasing evidence that antineoplastic drugs cause direct or indirect mitochondrial damage. Aside from affecting mitochondrial bioenergetics, mitochondrial DNA replication, mitochondrial oxidative and nitrosative stress, antineoplastic drugs can also cause cell death. Several studies have shown that dysregulation of mitochondrial dynamics contributes to antineoplastic drug-dependent cardiotoxicity. Therefore, understanding mitochondrial processes in cardiovascular toxicity is crucial to developing effective strategies to prevent myocardial harm or loss from various factors.
